# Chemical Kinetics of Metal Single Atom and Nanocluster Formation on Surfaces: An Example of Pt on Hexagonal Boron Nitride

**DOI:** 10.1021/acs.nanolett.3c01968

**Published:** 2023-08-18

**Authors:** Ilya Popov, Sadegh Ghaderzadeh, Emerson C. Kohlrausch, Luke T. Norman, Thomas J. A. Slater, Gazi N. Aliev, Hanan Alhabeadi, Andre Kaplan, Wolfgang Theis, Andrei N. Khlobystov, Jesum Alves Fernandes, Elena Besley

**Affiliations:** † School of Chemistry, University of Nottingham, University Park, Nottingham NG7 2RD, U.K.; ‡ School of Chemistry, Cardiff University, Cardiff CF10 3AT, U.K.; § School of Physics and Astronomy, University of Birmingham, Edgbaston, Birmingham B15 2TT, U.K.; ∥ Department of Physics, College of Science and Art, King Abdulaziz University, Rabigh 25732, Saudi Arabia

**Keywords:** single atom catalysts, point defects in 2D materials, nucleation kinetics, metal nanoclusters

## Abstract

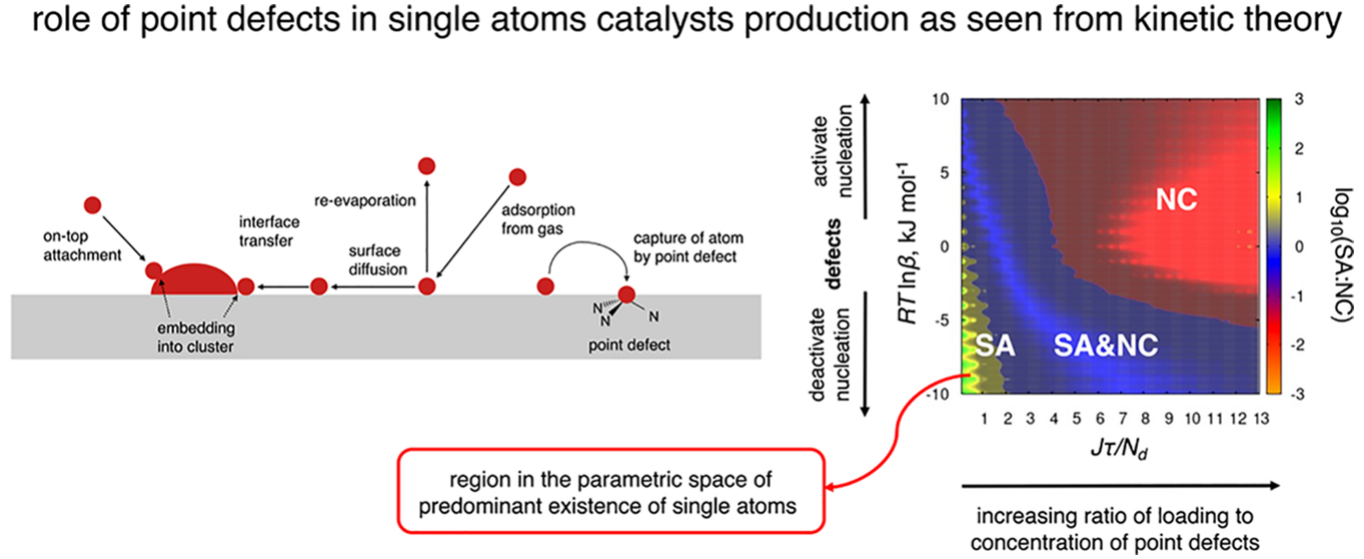

The production of atomically dispersed metal catalysts remains a significant challenge in the field of heterogeneous catalysis due to coexistence with continuously packed sites such as nanoclusters and nanoparticles. This work presents a comprehensive guidance on how to increase the degree of atomization through a selection of appropriate experimental conditions and supports. It is based on a rigorous macro-kinetic theory that captures relevant competing processes of nucleation and formation of single atoms stabilized by point defects. The effects of metal–support interactions and deposition parameters on the resulting single atom to nanocluster ratio as well as the role of metal centers formed on point defects in the kinetics of nucleation have been established, thus paving the way to guided synthesis of single atom catalysts. The predictions are supported by experimental results on sputter deposition of Pt on exfoliated hexagonal boron nitride, as imaged by aberration-corrected scanning transmission electron microscopy.

Efficient use of scarce precious metals in heterogeneous catalysis can be greatly improved with the development of thermally stable atomically dispersed catalysts. Single atom catalysts (SACs) often exhibit improved selectivity and activity in different industrially important reactions.^1,2^ Production methods of SACs typically employ either wet or dry synthetic routes.^3^ Wet synthesis of SACs includes adsorption-based methods such as facile adsorption^4^ and wetness impregnation,^5^ photoreduction,^6^ and ion exchange methods^7^ (see refs (3 and 8) for a comprehensive review). Dry routes are represented by variations of chemical and physical vapor deposition,^9−12^ thermally induced atom trapping^13−15^ and ball-milling methods.^16^ In these methods, single metal atoms, which are typically mobile and unstable on an ideal surface due to the high surface energy, get stabilized at anchoring sites such as functional groups, point defects, or other strong covalent binding sites.

Despite significant progress in production, characterization, and catalytic applications of SACs, the development of reliable methods for controlled synthesis of materials with increased fraction of metal surface atoms at a specified loading remains challenging. A well-known problem is coexistence, within the same material, of metal single atoms (SA) and nanoclusters (NCs) formed due to nucleation of mobile atoms.^3^ Selecting suitable experimental conditions to improve the SA:NC ratio requires a fundamental understanding of surface phenomena accompanying the formation of dispersed atoms and their influence on thermodynamics and kinetics of SACs production and stabilization. Although modern methods of analytical chemistry have had a tremendous impact on the development of our knowledge of the SACs structure and properties, comprehensive insights into the mechanisms underlying single atom formation are still lacking.^3,8^

Previous computational efforts supporting experimental work on SACs have been based mainly on density functional theory (DFT)^1,14,15,17−19^ and classical molecular dynamics simulations.^15,20^ High-throughput computational screening approaches to SACs design have been also proposed recently,^18,21^ mostly focusing on prediction of the catalytic activity. These approaches capture a small subset of key elementary processes involved in SACs production, while synthesis involves a great number of competing surface reactions, all influencing the outcome.

This work presents a predictive tool to guide the synthesis of metal single atom catalysts and nanoclusters on surfaces, which is based on a rigorous macro-kinetic formalism tested in the experiment. It captures relevant competing processes of stable single atom formation and homo- and heterogeneous nucleation of nanoclusters^a^ and defines key relations between experimental conditions and synthesis outcomes which are not accessible by direct measurements. This formalism can be applied readily to study the nucleation and formation of *any* metal single atoms stabilized by point defects on *any* surfaces. To validate the developed kinetic model in terms of accuracy of its predictions for the SA:NC ratio and nanocluster size, a flow of Pt atoms was produced and deposited on exfoliated hexagonal boron nitride (*h*-BN) using the magnetron sputtering technique^22^ and then imaged by aberration-corrected scanning transmission electron microscopy (AC-STEM). This metal atom deposition method is our preferred choice, as it includes processes essential to most SACs production techniques and involves a minimal number of subsidiary reactions, especially as compared to wet chemistry methods. It can be performed on a structurally well-defined support with a relatively small number of well-controlled experimental parameters, thus making it an ideal candidate for a direct comparison of conclusions drawn from theory and experiment.

We generalize the kinetic theory^23−28^ to describe the formation of stable single atoms on point defects, in addition to competing nucleation processes already captured by the theory. The resulting model describes the time evolution of the surface concentration of metal atoms and nanoclusters by a set of kinetic differential equations written in terms of statistically averaged parameters. This presents a macroscopic description of statistically relevant ensembles containing a large number of elementary reaction steps which occur on a long time scale, from seconds to hours. The effect of metal–support interactions and deposition parameters on the resulting SA:NC ratio has been analyzed focusing on the role of metal centers formed on point defects in the kinetics of nucleation. In the current approach, point defects on the surface have a fixed density, and the capture of metal atom by the defect is an irreversible process due to the high values of the binding energy.

Mobile single metal atoms are not attached to a point defect on the surface; they are unstable on the surface due to the excessive free energy^8,29^ and move freely until they nucleate and/or attach to the point defects. Measurements of the surface concentration of mobile single atoms cannot be performed directly, and importantly, mobile metal atoms do not contribute significantly to the estimation of the SA:NC ratio. The maximum concentration of single atoms that can be supported by a substrate is equal to the total of the density of point defects and the equilibrium concentration of mobile atoms that coexist with the solid phase of metal deposited on the surface. However, the equilibrium concentration of mobile atoms is orders of magnitude lower than the typical concentration of single atoms observed in our experiments (Supporting Information Section S1.1). The concentration of mobile atoms is therefore neglected, and the surface density of single atoms is attributed only to atoms captured by the point defects.

Figure 1a depicts a range of elementary reactions included in the kinetic model. Single metal atoms arrive at the surface from the gas phase with a constant rate, *J*. Once deposited onto the surface, metal atoms can participate in the following processes: (i) re-evaporation, a relevant atom depletion process which takes place when the binding energy of the atom to the surface is low; (ii) surface diffusion of atoms; (iii) capture of adsorbed atom by the point defect leading to the formation of an immobilized metal center; (iv) lateral attachment of metal atom to the growing nucleus of a NC; and (v) on-top attachment of metal atom directly from the gas phase to the growing NC. At room temperature, the surface mobility of NCs is negligible relative to the mobility of single atoms. The case of low surface submonolayer coverage has been considered here, and the coalescence processes occurring at the later stages of surface coating have been neglected.

**Figure 1:**
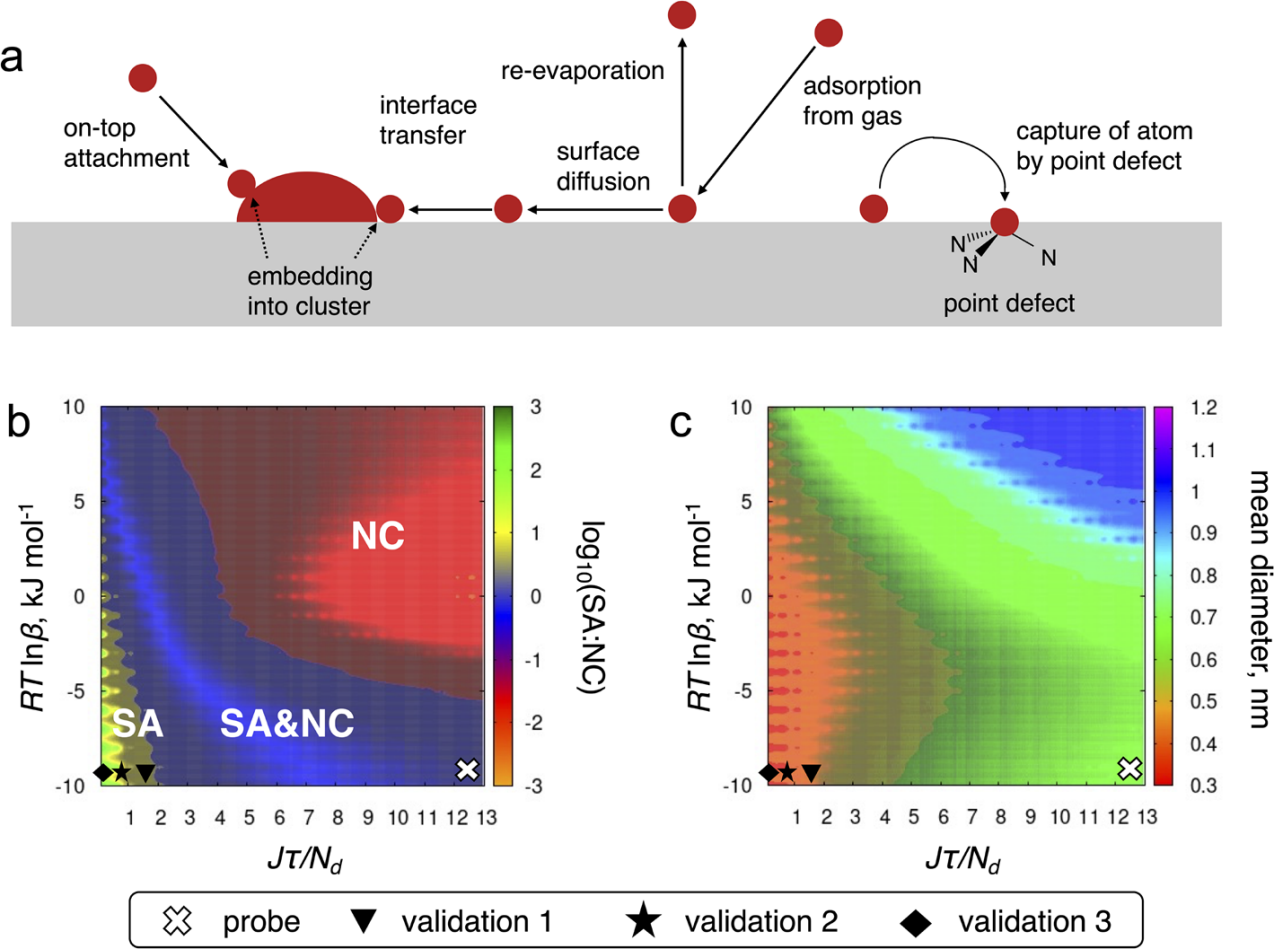
(a) Competing processes included in the kinetic nucleation theory. (b, c) Phase diagrams showing the dependence of the SA:NC ratio (logarithmic scale) and the mean diameter of NCs on the kinetic parameters involving the total loading of metal atoms, *J*τ, the density of point defects, *N*_d_, and the effective change in the nucleation barrier caused by the point defects, *RT* ln β. The white cross and black symbols on the phase diagrams correspond to the probe and validation experiments; their approximate positions on the *J*τ/*N*_d_ axis are 12.55, 1.55, 0.35, and 0.07.

Within the assumptions of kinetic nucleation theory, metal nanoclusters can be formed by (i) homogeneous nucleation through the attachment of a mobile surface atom to a growing nucleus on a defect-free part of the support and (ii) heterogeneous nucleation where a single atom gets attached to an immobilized metal center created on a point defect. Because of the different chemical nature, these mechanisms are characterized by different rates of single atom attachment. We denote the surface concentration of clusters formed in homogeneous nucleation as *n*_*i*_ (*i* is the number of atoms in a cluster), while clusters formed on point defects are labeled as *f*_*i*_. The evolution of the surface nucleation process in time can be described by the following set of differential equations:
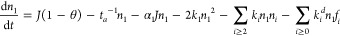
1
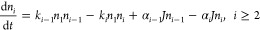
2
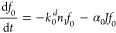
3
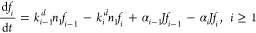
4where the fractional surface occupancy is given by
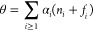
5In these equations, *n*_1_ corresponds to the surface concentration of mobile single metal atoms, *f*_0_ is the concentration of point defects without attached metal atoms, and *f*_1_ is the concentration of single metal atoms trapped by the point defects.

Equation 1 describes processes involving a deposited metal atom as the time evolution of the surface concentration of single atoms, *n*_1_. It includes the rates of all processes shown in Figure 1a and those described earlier in the text. The first term in eq 1 corresponds to the flow of atoms to an unoccupied part of the surface followed by re-evaporation of adatoms, on-top attachment directly from the gas, formation of a dimer from two adatoms, and, finally, the last two terms describe the lateral attachments of metal atoms to nanoclusters growing on a defect-free part of the surface and on point defects, respectively. Equations 1 and 4 are solved with the initial conditions *f*_0_(0) = *N*_d_, *n*_*i*_(0) = 0, and *f*_*i*_(0) = 0 for *i* ≥ 1. If deposition occurs over time τ, then the observed ratio of single atoms to nanoclusters can be estimated as
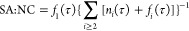
6The kinetic parameters α_*i*_, *t*_*a*_, *k*_*i*_, and *k*_*i*_^*d*^ are time-independent. Parameters α_*i*_, containing the values of the surface area occupied by the clusters consisting of *i* atoms, can be calculated for any metal and shape of the cluster as described in Supporting Information Section S1.2. Here, we assume the two-dimensional shape of clusters as the most reasonable approximation to the experimental AC-STEM images shown in Figure S5. The lifetime of mobile adatoms can be calculated from the desorption energy, *E*_des_, as
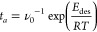
7where ν_0_ ≈ 10^13^ s^–1^ is the standard vibrational frequency (see for example ref (30)). From the value of the desorption energy of Pt on *h*-BN calculated by DFT,^31^ the lifetime can be estimated as *t*_*a*_ ≈ 10^12^ s. This implies that re-evaporation of Pt atoms is not a relevant process in this case and can be neglected. The rate constants *k*_*i*_ and *k*_*i*_^*d*^ correspond to the lateral attachment of metal atoms to NCs growing on ideal parts of the surface and point defects, respectively. These processes yield the most significant contributions to nanocluster growth.

In homogeneous nucleation, the lateral attachment is not an elementary process as it includes three principal stages: atom diffusion toward the NC, interface transfer, and embedding of metal atom into the NC structure. The relative rates of these processes determine the kinetic regime of nucleation and the corresponding mathematical expressions for the rate of attachment.^25,27^ If the energy barrier to surface diffusion of metal atoms is high (higher than ca. 40–50 kJ mol^–1^), diffusion becomes a limiting step, and nucleation proceeds in the diffusive regime. This is the case for Pt on *h*-BN as DFT calculations estimate the diffusion barrier of *E*_diff_ = 74 kJ mol^–1^,^31^ indicating that the attachment process and NCs growth are governed by the surface diffusion of single metal atoms. A slightly lower value of the diffusion barrier used in this work, *E*_diff_ = 55 kJ mol^–1^, leads to a good agreement with the experimentally observed width of the NCs size distribution shown in Supporting Information Section S1.3. It lies within a typical range of the values for the diffusion barrier predicted by different DFT setups. The DFT diffusion barrier of ref (31) does not account for dispersion corrections to the total energy, which tend to lower the difference between the transition and ground states, and it is calculated for a monolayer of *h*-BN, whereas our experimental setup produces multilayer nanosheets.^32^

Generally, the mobility of atoms on a surface is characterized by the diffusion coefficient
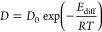
8where the pre-exponential factor can be evaluated as^33^
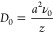
9*a* is the distance between two nearest energy minima on the support lattice, and *z* is the coordination number of the energy minimum. DFT calculations^31^ confirm that for Pt on *h*-BN the strongest adsorption site is top of N atom. This gives the values of *a* = 0.2504 nm and *z* = 6, resulting in *D*_0_ = 1.045 × 10^–7^ m^2^ s^–1^, which is in good agreement with previously reported values of *D*_0_ for similar processes on different substrates. For example, experimental values of *D*_0_ obtained using scanning tunneling microscopy for Pt^34^ and Ag^28^ on Pt(111) are 1.283 × 10^–7^ and 1.797 × 10^–6^ m^2^ s^–1^, respectively. Another DFT analysis of metal mobility on α-Al_2_O_3_(0001) surface^35^ yields the values of *D*_0_ in the range 10^–6^–10^–8^ m^2^ s^–1^ depending on the metal. *Ab initio* molecular dynamics (AIMD) simulations for Ag and Cu on graphite give *D*_0_ = 3.17 × 10^–7^ m^2^ s^–1^ and *D*_0_ = 1.44 × 10^–7^ m^2^ s^–1^, respectively.^36^

In the diffusive regime, the rate constant for nucleation on a defect-free area of the surface is given by
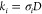
10where σ_*i*_ is a slowly varying function of the number of atoms.^25^ The rate constants, *k*_*i*_^*d*^, describing the attachment processes occurring on metal atom occupying a point defect are scaled as
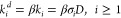
11If the scaling factor β > 1, then surface point defects activate the nucleation, and if β < 1, they deactivate the process. Chemically, the value of β is determined by the difference in the energy barriers describing the attachment on a defect and on the ideal surface as
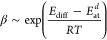
12where *E*_at_^*d*^ is the barrier to the attachment of an adatom to the metal center formed on a point defect.

At room temperature, *RT* ≈ 2.5 kJ mol^–1^ so that β is expected to be significantly different from unity, i.e., β ≫ 1 or β ≪ 1. For any type of point defect, it would be difficult to estimate the value of the scaling factor β directly from experiment; however, it can be obtained computationally. It would require calculations of a few barriers corresponding to the consequent attachments of adatoms to the metal center formed on a point defect and comparison with the barrier for diffusion. Here, we provide a few qualitative assumptions regarding the behavior of the scaling parameter β for different types of point defects. Deactivation of nucleation (β < 1) is possible when the interaction of metal atom with point defect yields significant change in the oxidation state and electronic structure of the atom. This typically happens when strong covalent bonds are formed between metal atom and point defect, for example, in the case of vacancies and substitutional atoms with high electronegativity such as oxygen. At the same time, foreign atoms or clusters (e.g., organic matter) adsorbed on the surface will activate heterogeneous nucleation, resulting in β > 1.

In summary, the kinetics of a nucleation process and resulting composition of the obtained ensemble can be determined by the following parameters: the flow rate of metal atoms to the surface (*J*), time of deposition (τ), initial concentration of the defects (*N*_d_), diffusion coefficient (*D*), and scaling parameter (β). The first two kinetic parameters can be controlled within an experimental setting, and the remaining three parameters describing the quality of support surface and the nature of metal–support interactions can be obtained computationally. In the context of the production of atomically dispersed SAC materials, it is important to understand how the SA:NC ratio depends on the parameters of the kinetic model.

We conclude that the SA:NC ratio depends weakly on diffusion coefficient *D*, which mostly affects the shape of the NCs size distribution function (the role of *D* is discussed in Supporting Information Section S1.4). The effect of the other four kinetic parameters on the SA:NC ratio is represented by the phase diagram, shown in Figure 1b, which demonstrates that the manifold of the parameters is divided into three distinct areas: (i) the green-yellow region of predominant existence of single atoms, (ii) the red-orange area of predominant existence of nanoclusters, and (iii) the blue phase, where SA and NC coexist in comparable quantities (where ca. 0.25 ≤ SA:NC ≤ 4.0). For the SACs production, the green region marks the most desirable outcome, where the formation of nanoclusters is almost completely suppressed. The phase diagram in Figure 1b gives a clear guidance on how to improve SACs production by selecting an appropriate support and tuning experimental parameters of the magnetron sputtering deposition process.

In experiment, we optimize the magnetron sputtering setup in order to reach the region below the critical boundary in the parameter space separating blue and green-yellow phases in Figure 1b. In the investigated parameter domain, this boundary has almost linear behavior and it can be approximated as
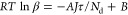
13with *A* = 5.91 kJ mol^–1^ and *B* = 3.05 kJ mol^–1^. This approximation has been used to estimate the critical loading that can be achieved for a given support with the known concentration and type of point defects. Vice versa, if a particular load is desired, it can also predict which type of point defects and in what concentration is required. Additionally, we plot the dependence of the mean diameter of NCs on the same kinetic parameters (Figure 1c) obtained for a given value of the diffusion coefficient and deposition time τ = 1 s. For *J*τ/*N*_d_ < 5, the range of diameters form a valley in the parameter space, where the sizes of NCs are quite small, of the order of 0.35–0.55 nm, whereas higher ratios of the loading to the concentration of defects yield larger sizes of NCs.

The proposed general kinetic analysis has been validated by the magnetron sputtering experiments where Pt was deposited on the *h*-BN support, and for the produced samples, the values of the SA:NC ratio and mean diameter of NCs were extracted (experimental details are given in Supporting Information Sections S2.1 and S2.2). A direct comparison of experimental results with theoretical predictions would require full structural characterization of the support, which represents a significant challenge to experiment. In the absence of precise knowledge of the types and concentration of point defects present on the *h*-BN support, a probe experiment has been first undertaken aimed at extracting a reasonable range of the two unknown parameters of the kinetic model, namely, β defining the type of defects and *N*_d_ determining their concentration.

In the probe experiment, an exfoliated *h*-BN sample was exposed to the flow of Pt atoms with the rate estimated at *J* = 1.77 ± 0.40 nm^–2^ s^–1^ for 1 s (see Supporting Information Sections S2.1 and S2.2), and the electron microscopy analysis confirmed coexistence of single atoms with nanoclusters as shown in Figure 2. The SA:NC ratio was estimated to range from 0.8 to 1.2 across different areas in the image, indicating that this experimental setup corresponds to the blue region of the phase diagram shown in Figure 1b. The experimental mean diameter of NCs is determined to be 0.7 ± 0.3 nm.

**Figure 2:**
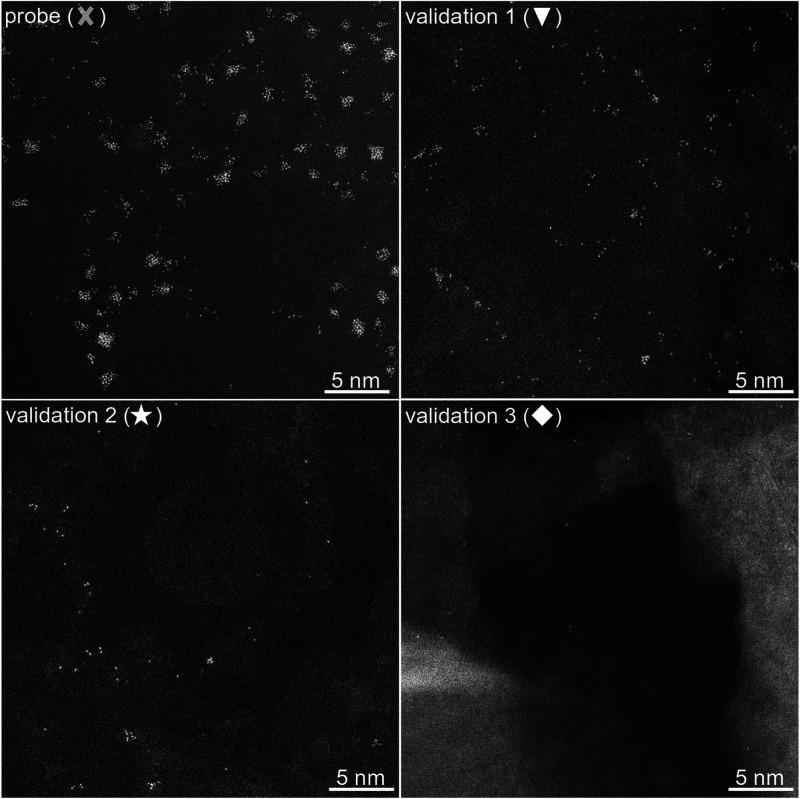
AC-STEM images of four samples with different loading of Pt on *h*-BN: *J* = 1.77 nm^–2^ s^–1^ in probe experiment, *J* = 0.22 nm^–2^ s^–1^ in validation 1, *J* = 0.05 nm^–2^ s^–1^ in validation 2, and *J* = 0.01 nm^–2^ s^–1^ in validation 3 experiments. The time of deposition is equal to 1 s in all cases.

From these data, the upper boundary for the concentration of defects (*N*_d_ ≤ *J*τ/5) and for the values of β (*RT* ln β ≤ −5 kJ mol^–1^ and β ≤ 0.14) were deduced. Additionally, analysis of the phase diagram in Figure 1b in the wider range of parameters (see Supporting Information Section S1.5) shows that the blue area is bound to the left by the values of *J*τ/*N*_d_ ≤ 14. It gives a lower boundary for the defect concentration of *N*_d_ ≥ *J*τ/14. Using the experimental values of *J* and deposition time τ = 1 s, we estimate the surface concentration of point defects as 0.09 ≤ *N*_d_ ≤ 0.40 nm^–2^, which agrees very well with the value recently reported in ref (37) where the total surface density of point defects in exfoliated *h*-BN was measured as *N*_d_ = 0.14 nm^–2^. A vast majority of these defects (85%) corresponds to the vacancies in which boron atom is removed from the lattice, while vacancies with missing nitrogen atom and divacancies accounted for the remaining 15%. Note that photoluminescence (PL) spectrum of the *h*-BN samples used in this work exhibits similar peaks to the PL spectrum reported in ref (37). This indirectly indicates the presence of a comparable concentration of point defects responsible for the emission in the visible range (Supporting Information Section S2.3).

The results of the probe experiment, having a defect concentration similar to the previously reported experimental value of *N*_d_ = 0.14 nm^–2^, are marked by the white cross on the phase diagrams in Figure 1. As follows from the phase diagram in Figure 1b, the SA:NC ratio can be further increased by decreasing the value of *J*τ/*N*_d_. The simplest way to do this without modifying the support is to decrease the loading. To validate this conclusion, three additional samples were produced by magnetron sputtering (Supporting Information Sections S2.1 and S2.2) with gradually decreasing loading values, which are tabulated in Table 1. The AC-STEM images of these samples are presented in Figure 2. The results of the validation experiments are also marked in the phase diagram in Figure 1b by the black symbols.^b^

**Table 1:** Experimental and Theoretical Values of the SA:NC Ratio and Mean Diameter of Nanoclusters for Four Different Loadings of Pt on *h*-BN^a^

		SA:NC ratio		NC mean diameter (nm)	
	expt loading (nm^–2^)	expt	theory	expt	theory
probe	1.77 ± 0.40	1.0 ± 0.2	0.3	0.7 ± 0.3	0.7
validation 1	0.22 ± 0.08	1.5 ± 0.5	1.7–16.5	0.6 ± 0.2	0.4–0.5
validation 2	0.05 ± 0.03	4.8 ± 2.5	19.8–200.0	0.6 ± 0.1	0.4
validation 3	0.01 ± 0.01	only SA	129.1–411.2		

a Experimental data were obtained from the analysis of a series of the AC-STEM images as described in Section S2.2 of the Supporting Information.

The predicted theoretical values of the SA:NC ratio and NCs mean diameter are compared with experimental data in Table 1, which shows that the kinetic theory provides excellent agreement for the NCs average size and captures the trend in variation of the SA:NC ratio. The latter is particularly encouraging considering the limited statistics provided by the AC-STEM images.

In summary, a chemical kinetic model has been applied to investigate the production of SACs on surfaces containing point defects and to reveal general trends describing the dependence of the SA:NC ratio on the key kinetic parameters such as metal loading and the chemical nature and concentration of point defects. These predictions provide useful guidance for the choice of experimental conditions and the design of supports in the targeted synthesis of SACs. As a proof of concept, sputter deposition of Pt on *h*-BN was performed to probe the predicted trends.

## Supporting Information Available

Details of theoretical analysis; equilibrium concentration of surface mobile atoms; parameters α_*i*_; size distribution function; SA:NC dependence on the diffusion parameter; phase diagram of SA:NC in a wider range of kinetic parameters; experimental methods; Pt deposition onto hexagonal boron nitride; experimental determination of Pt coverage onto hexagonal boron nitride (*h*-BN); photoluminescence (PL) measurements (PDF)

## Footnotes

a Nanoclusters include all particles, which are not single atom, starting from dimers and their size is limited to 1 nm. The presented chemical kinetics model is general and not limited to a particular size range, and it can be applied to bigger nanoparticles.

b These positions are determined without taking into account error bars in the experimental values of loading and must be taken as approximate locations.
