# Supplementary material for: Chemical Kinetics of Metal Single Atom and Nanocluster Formation on Surfaces: An Example of Pt on Hexagonal Boron Nitride
Source: Nano Lett. 2023 Aug 18;23(17):8006–12. doi: 10.1021/acs.nanolett.3c01968 (PMC10510580; doi:10.1021/acs.nanolett.3c01968)
Supplement: Supplementary file 1 — nl3c01968_si_001.pdf [file nl3c01968_si_001.pdf]

Supporting Information.
Chemical Kinetics of Metal Single Atom and Nanocluster Formation on Surfaces: an Example of Pt on Hexagonal Boron Nitride

Ilya Popov,[†] Sadegh Ghaderzadeh,[†] Emerson C. Kohlrausch,[†] Luke T. Norman,[†] Thomas J. A. Slater,[‡] Gazi N. Aliev,[¶] Hanan Alhabeadi,[¶,§] Andre Kaplan,[¶] Wolfgang Theis,[¶] Andrei N. Khlobystov,[†] Jesum Alves Fernandes,[*,†] and Elena Besley[*,†]

†*School of Chemistry, University of Nottingham, University Park, Nottingham NG7 2RD, UK*

‡*School of Chemistry, Cardiff University, Cardiff, CF10 3AT, UK*

¶*School of Physics and Astronomy, University of Birmingham, Edgbaston, Birmingham B15 2TT, UK*

§*Department of Physics, College of Science and Art, KAU, Rabigh 25732, Saudi Arabia*

E-mail: Jesum.AlvesFernandes@nottingham.ac.uk; Elena.Besley@nottingham.ac.uk

S1 Details of theoretical analysis

S1.1 Equilibrium concentration of surface mobile atoms

Surface concentration of metal adatoms N_{eq} corresponding to the equilibrium vapor pressure p_{eq} of metal deposited on a support can be estimated as follows:[1]

$$N_{eq} = \frac{p_{eq} t_a N_a}{\sqrt{2\pi MRT}}, \tag{1}$$

where M - is atomic mass of adatom, N_a is the Avogadro constant and $t_a \approx 10^{12}$ [s] is the life-time of adatoms described in the main text. Although direct measurement of the equilibrium vapor pressure of Pt deposited on h-BN is not available in the literature, it would be reasonable to assume that its value is of the same order of magnitude as the vapor pressure for solid Pt. The latter can be approximated in the range from 298K to the melting point by the following empirical equation:[2]

$$\log p_{eq}\,[\mathrm{atm}] = 4.882 - 29387T^{-1} + 1.1039\log T - 0.4527T^{-3}, \tag{2}$$

where the temperature is in K. Inserting the values yields $N_{eq} \sim 10^{-52}$ [m^{-2}] for the room temperature, which is negligible.

S1.2 Parameters α_i

Surface area occupied by a two-dimensional cluster consisting of i atoms can be calculated as

$$\alpha_i = \frac{i}{\rho_m}, \tag{3}$$

where $\rho_m = {}^2/_{\pi d_m}$ is a surface density of metal atoms in the hexagonal monolayer and d_m is a length of intermetallic bond, which can be calculated from the lattice parameter of

fcc structure as $d_m = {}^a\!/\!\sqrt{2}$. Note, that the area calculated in this way is not directly related to the one, which can be observed by AC-STEM imaging, since it includes the surrounding space, getting into which the metal atom will bind to the cluster. This approximate equation works well starting from $i \geq 4$. For single atoms, dimers and trimers we evaluate α_i using the following equation

$$\alpha_i = \pi r_i^2, \tag{4}$$

where radii are $r_1 = d_m$ and $r_2 = r_3 = 2d_m$.

In conclusion of this Section, we note that in the experimental conditions described here the surface occupation is rather low (see the AC-STEM image from the main text) suggesting that on-top impinging rate is a minor contribution to the nanoclusters growth compared to the lateral attachments of adatoms. To support this statement we plot the ratio of total rates of lateral and on-top attachments as a function of deposition time (Figure S1). From this graph one can see that up to $\tau = 1$s lateral attachments completely prevail over on-top ones. It means, that the choice of α_i parameters has a minor effect on the conclusions of the main text. Therefore, we do not pay significant attention to these parameters in the main text.

S1.3 Size distribution function

Comparison of theoretical NCs size distribution calculated for $E_{diff} = 55$ kJ $\cdot$ mol^{-1} with experimental data following from the analysis of AC-STEM images for the "probe" experiment (see the main text). As one can see, this value of the diffusion barrier allows to reproduce the width of the size distribution. The latter depends significantly on the diffusion barrier, therefore, we use the value of 55 kJ $\cdot$ mol^{-1} in the main text, which is slightly lower than the one obtained by DFT calculations (see discussion in the main text).

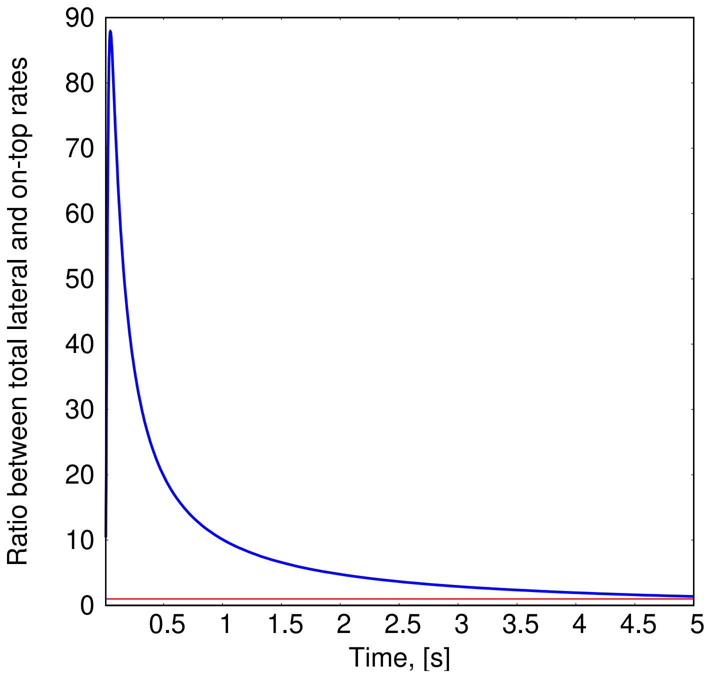

Figure S1: Ratio of total rates of lateral and on-top attachments (blue line) as compared to unity (red line) as a function of deposition time.

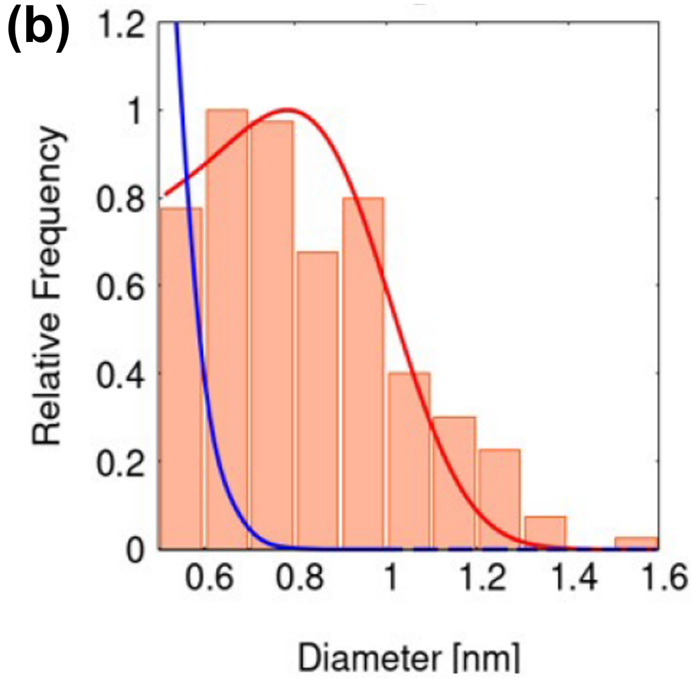

Figure S2: An example of the size distribution functions for the "probe" experiment from the main text. Orange bars correspond to the data obtained from the experimental AC-STEM images, while curves - to the results of the kinetic theory (obtained for $\beta = 0.04$, $RT \ln \beta \approx -8$ kJ $\cdot$ mol^{-1}). Here, red curves correspond to NCs formed on the clear surface, while blue curves - to NCs formed on the point defects.

S1.4 SA:NC dependence on the diffusion parameter

Let us demonstrate the behaviour of SA:NC ratio for different values of the diffusion parameter D for a particular case $\beta = 1$ allowing an analytical treatment. To do that, we assume low surface occupation by neglecting on-top attachment terms (see discussion in the previous Section). In addition, we neglect very slow i dependence of parameters σ_i[3] be setting $k_i = k = \sigma D$.

In the case of $\beta = 1$ nucleation on the point defects completely prevails over nucleation on ideal parts of the surface, therefore, taking into account all assumptions, we obtain the following system of equations:

$$\frac{dn_1}{dt} = J - kN_d n_1, \tag{5}$$

$$\frac{df_0}{dt} = -kn_1 f_0, \tag{6}$$

$$\frac{df_1}{dt} = kn_1 \left(f_0 - f_1\right), \tag{7}$$

$$\mathrm{SA:NC} = \frac{f_1(\tau)}{N_d - f_1(\tau) - f_0(\tau)}. \tag{8}$$

Solutions for $n_1(t)$ and $f_0(t)$ with initial conditions $n_1(t) = 0$ and $f_0(0) = N_d$ are straightforward:

$$n_1(t) = \frac{J}{kN_d}\left[1 - \exp\left(-kN_d t\right)\right], \tag{9}$$

$$f_0(t) = N_d \exp\left[-s(t)\right], \tag{10}$$

where

$$s(t) = \frac{Jt}{N_d}\left(1 - \frac{1 - \exp(-kN_dt)}{kN_dt}\right) \tag{11}$$

To solve the equation for $f_1(t)$ let us introduce a new function $y(t) = {}^{f_1(t)}/_{f_0(t)}$. Then the equation transforms into

$$\frac{dy}{dt} = kn_1, \qquad y(0) = 0, \tag{12}$$

which finally yields

$$y(t) = \frac{f_1(t)}{f_0(t)} = s(t). \tag{13}$$

The ratio SA:NC can be then expressed through $s(\tau)$ as:

$$\mathrm{SA}:\mathrm{NC} = \frac{s(\tau)\exp[-s(\tau)]}{1 - [1 + s(\tau)]\exp[-s(\tau)]}. \tag{14}$$

Let us now analyse $s(\tau)$ as a function of D. From its functional form, one obtains

$$s(\tau) \approx \frac{J\tau}{N_d}, \qquad \sigma DN_d\tau \gg 1 \tag{15}$$

$$s(\tau) \approx \frac{\sigma JD\tau^2}{2}, \qquad \sigma DN_d\tau \ll 1 \tag{16}$$

In the first case $s(\tau)$ and, hence, the SA:NC ratio do not depend on the diffusion parameter D. In the second case $\mathrm{SA}:\mathrm{NC} \sim D^{-1}$. Taking the smallest value of the defects concentration considered in the extended phase diagram Figure S3 ($N_d = {}^{J\tau}/_{30}$), one obtains that the first case is satisfied when the diffsuion barrier is lower than ≈ 60 [$\mathrm{kJ} \cdot \mathrm{mol}^{-1}$]. This is the case for our system, therefore, one can safely neglect dependence of the SA:NC ratio on D. In addition, we note that according to DFT study[4] Pt has the highest barrier of diffusion on *h*-BN among all investigated metals, which makes it possible to conclude, that

the weak dependency on D will be satisfied for other metals on h-BN as well.

S1.5 Phase diagram of SA:NC in a wider range of kinetic parameters

Phase diagram showing dependence of the SA:NC ratio on ${}^{J\tau}/{}_{N_d}$ and $RT \ln \beta$ in a wider range of values of parameters is shown in Figure S3. Note, that the blue area is bounded on the left. This allows us to estimate a range of the point defects concentration discussed in the main text.

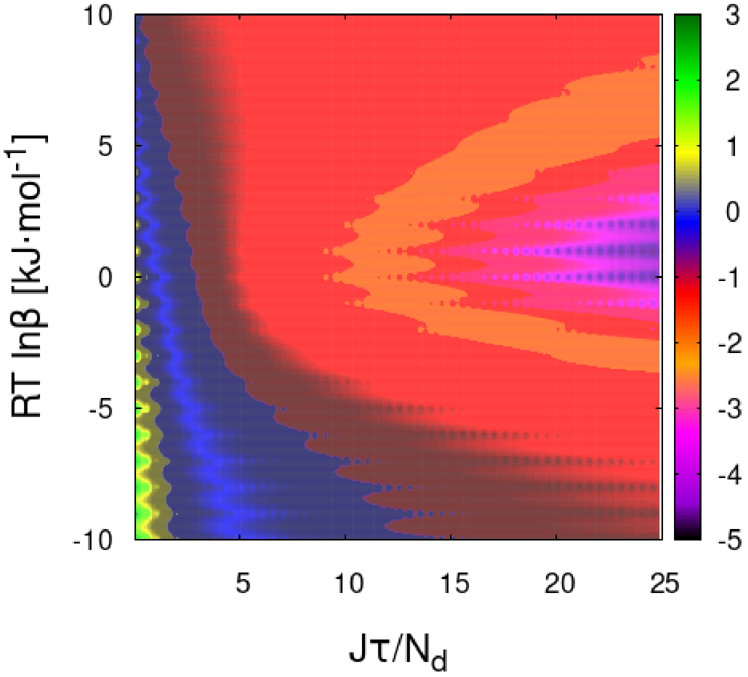

Figure S3: Decimal logarithm of the SA:NC ratio as a function of kinetic parameters ${}^{J\tau}/{}_{N_d}$ and $RT \ln \beta$ in a wider range of their values.

S2 Experimental Methods

S2.1 Pt deposition onto hexagonal boron nitride

Hexagonal boron nitride (*h*-BN) nanosheets were produced by exfoliation of bulk crystals in isopropyl alcohol[5] and then used as a support. To prepare the support, *h*-BN powder (200 mg) was dispersed in isopropyl alcohol (400 mL), sonicated for 15 min, then dropcasted (7 drops) onto a holey carbon Cu TEM H7 finder grid (FG) and/or non-finder (conventional) grid (NF), and left to air dry for 30 mins.

Magnetron sputtering depositions took place using a bespoke built AJA magnetron sputtering system. The platinum (99.99% purity) target was purchased from Kurt J Lesker. The TEM grids were secured on a flat disc support and then placed in a load lock and pumped down to $5.0 \cdot 10^{-6}$ Torr for 0.5 hour, and then transferred into the main chamber and then pumped down to $6.0 \cdot 10^{-7}$ Torr for 0.5 hour. The work pressure (Argon 99.9995%) was 3 mTorr, current applied of 50 mA, 1 sec deposition time. The samples were positioned at different distance from target in order to obtain different levels of Pt loading (coverage) on *h*-BN as shown in the Figure S4 (see also Ref.[6]).

S2.2 Experimental determination of Pt coverage onto Hexagonal boron nitride (*h*-BN)

Pt species coverage onto *h*-BN was determine using two different techniques by aberration corrected Scanning Transmission Electron Microscopy (ac-STEM) where Pt was deposited onto *h*-BN powder dispersed on TEM grids and to validate it we also determine the Pt coverage by Inductively Coupled Plasma Optical Emission spectroscopy (ICP-OES) where Pt was deposited on filter paper.

Pt species coverage on *h*-BN was determined by analysing ac-STEM images with a custom python program. As an example, the image in Figure S5 is used to describe all the steps used

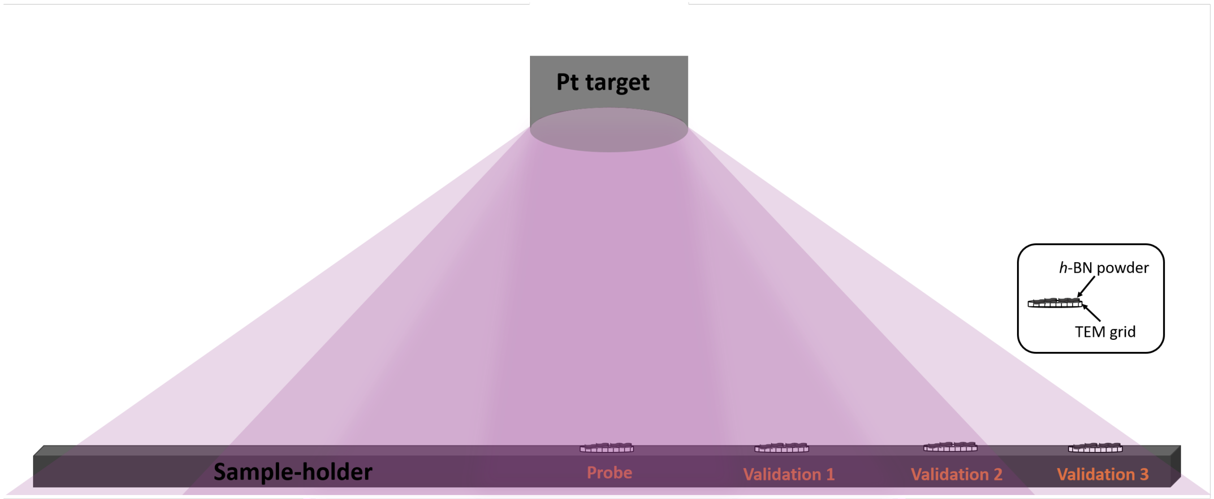

Figure S4: Illustrative scheme of one pot Pt atoms deposition onto *h*-BN by magnetron sputtering generating different Pt loading for each sample. Pt qualitative coverage decreases as the sample is positioned farther from the target. The quantitative experimental Pt coverage results are presented in the next Section.

to determine the Pt coverage and SA:NC ratio. Pt atoms without additional Pt atoms within an approximately 0.5 nm radius were labelled as single Pt atoms. Using this approach, a SA:NC ratio of 1.0 was found for the image in Figure S6.

For Pt coverage on *h*-BN the number of single atoms and atoms in dimers and trimers were directly counted. For larger NCs, the number of atoms in the cluster was calculate by the NC's integrated background-subtracted image intensity divided by the average single Pt atom intensity. The resulting total number of atoms in the image (including atoms, dimers and lager NCs) was divided by the area of the *h*-BN substate in the respective image as illustrated in Fig S6.

To validate this approach to determine Pt coverage, ICP-OES measurements was carried out using filter paper as support as the amount of Pt deposited onto *h*-BN dispersed on TEM grid would be not be detectable via ICP-OES. A filter paper with a diameter of 5.08 cm was placed in the homogenous Pt deposition rate region (Figure S4 and Ref.[6]) as the probe sample to ensure that the coverage obtained in the filter paper could be correlated with the Pt coverage in *h*-BN dispersed in TEM grid.

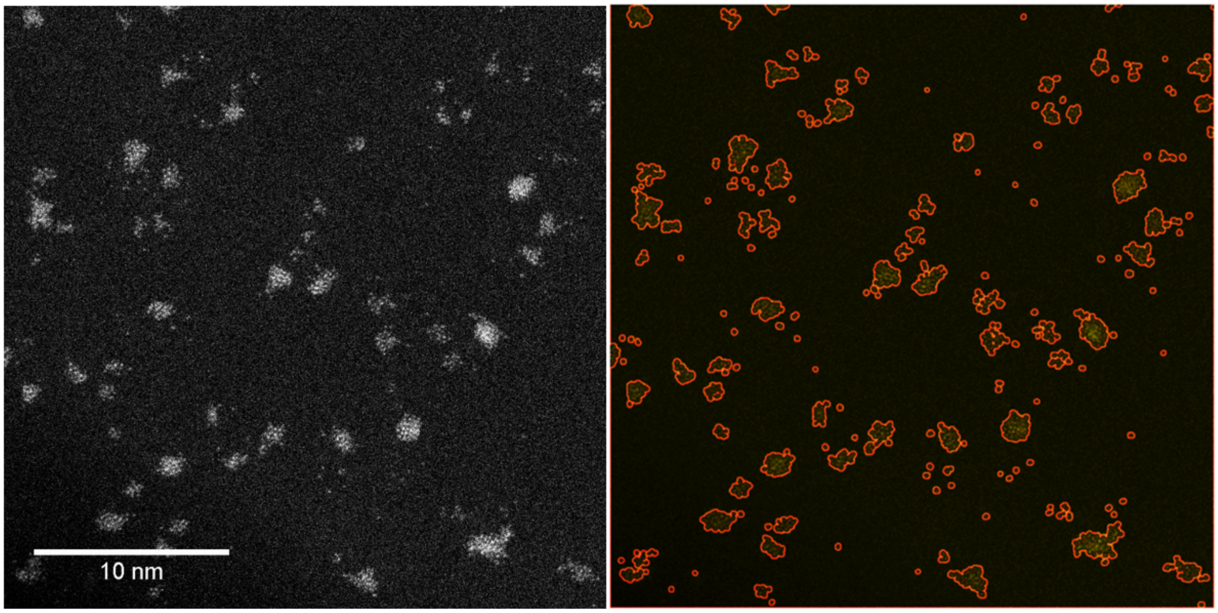

Figure S5: (a) An example of HAADF-STEM electron microscopy image of the probe sample used to calculate the Pt coverage onto h-BN and the SA:NC ratio. (b) Identification of single atoms, dimers and larger NCs.

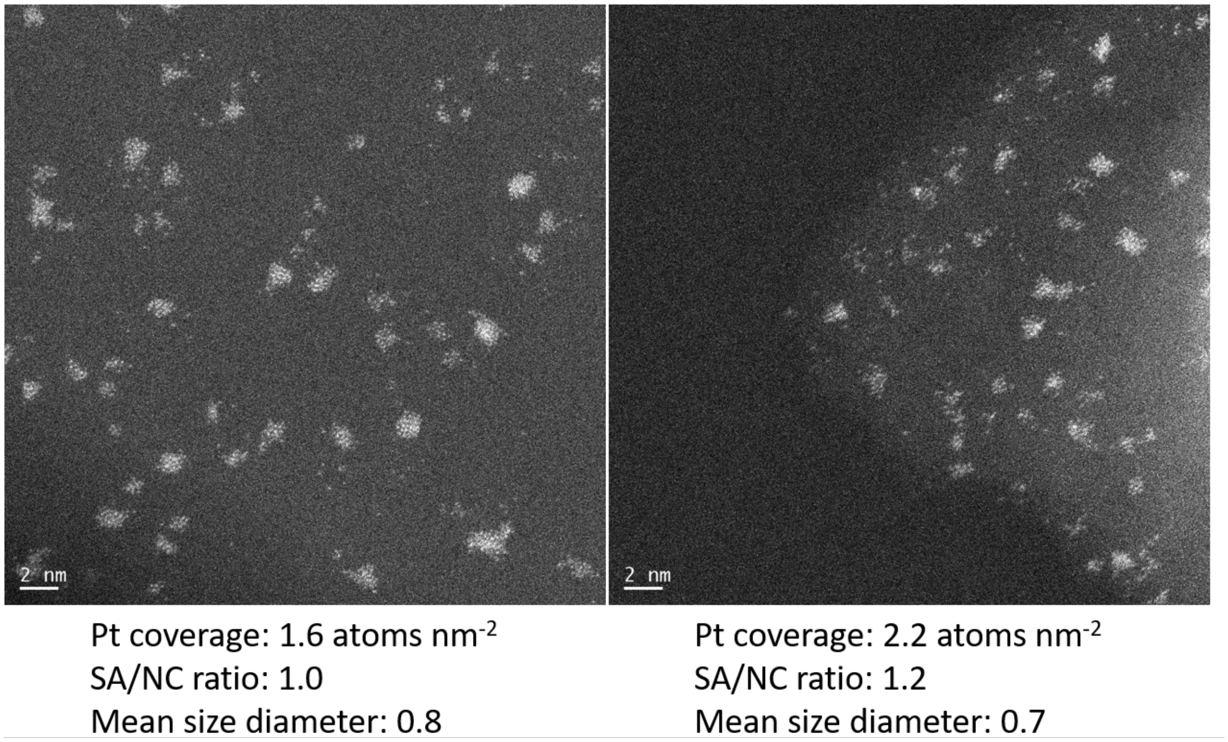

Figure S6: Electron microscopy images of the probe sample used to calculate the average of Pt coverage onto h-BN, SA:NC ratio and NCs mean diameter. Pt coverage onto h-BN was found 1.77 ± 0.4 atoms nm^{-2}; SA:NC 1.0 ± 0.2 and mean size diameter 0.7 ± 0.3 nm.

The work pressure (Argon 99.9995%) was 3 mTorr, current applied of 50 mA, work distance of 90 mm and rotation at 120 rpm to ensure the deposition homogeneity (see details in Table S1 and Figure S7a). After the deposition, 80 mg (6 cm^2) of a sample was accurately weighted in a flask, per each sample. 2 mL of a solution of one part concentrated nitric acid and three parts concentrated hydrochloric acid (aqua regia) was added to each flask. After 24h, the metals were completely dissolved in the solution and then it was centrifuged three times to remove all remaining particles of the filter paper. Afterwards, the obtained solutions were diluted and transferred into a 10 ml volumetric flask using a 5% HCl solution (% v/v in ultra pure water). The volumetric flasks were filled to the mark with this 5% HCl solution and then analysed using calibration curves with the appropriate Pt calibration standards. Each sample was measured in 2 different wavelengths, with high sensitivity. Wavelengths used for the determination of Pt content in solution were 265 nm and 214 nm. As can be observed in the Figure S7b, the Pt concentration increases linearly as the deposition time increases (see Table S2), therefore we can extrapolate this calibration for lower and/or higher deposition times as the sample is positioned in the homogeneous Pt deposition rate region.

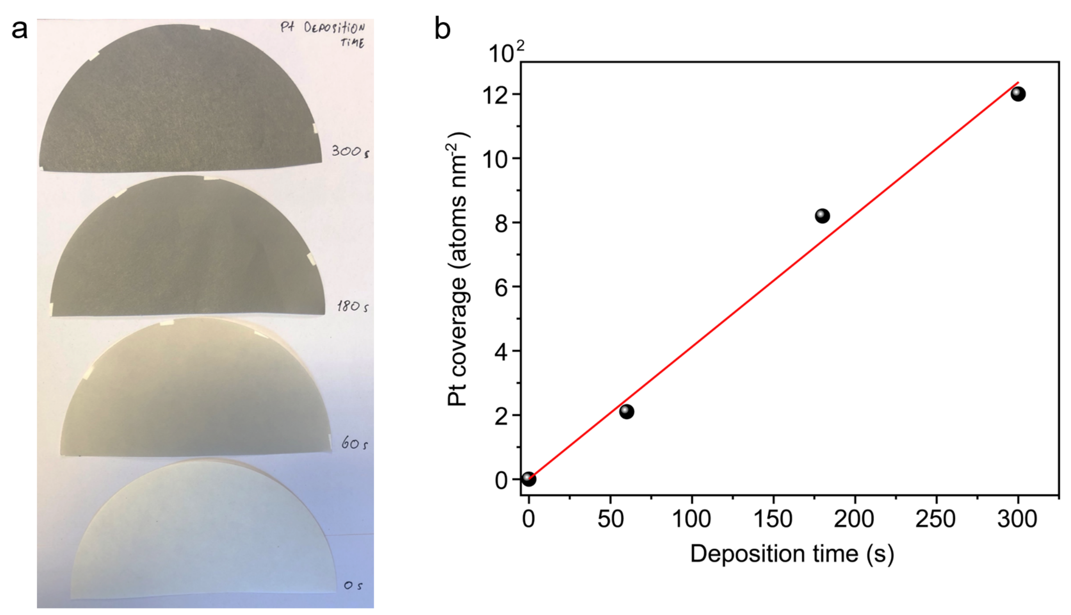

Figure S7: Pt atoms deposition on filter paper. (a) Photo of Pt deposited on filter paper, (b) Linearly dependence of Pt coverage in filter paper as deposition time increases.

Table S1: Summary of magnetron sputtering parameters for Pt deposition onto filter paper and ICP-OES results.

Deposition time (s)	Current (mA)	Voltage (V)	Power (W)	ICP-OES (atoms nm^{-2})
60	50	282	13	210
180	50	282	13	820
300	50	290	14	1200

Table S2: Summary of the Pt coverage onto *h*-BN experimentally determined using AC-STEM and ICP-OES. Values correspond to Pt coverage for 1s deposition (atoms nm^{-2}).

	AC-STEM	ICP-OES
Sample	5 images average	Calibration curve extrapolation
Probe	1.8 ± 0.4	4.0 ± 0.5

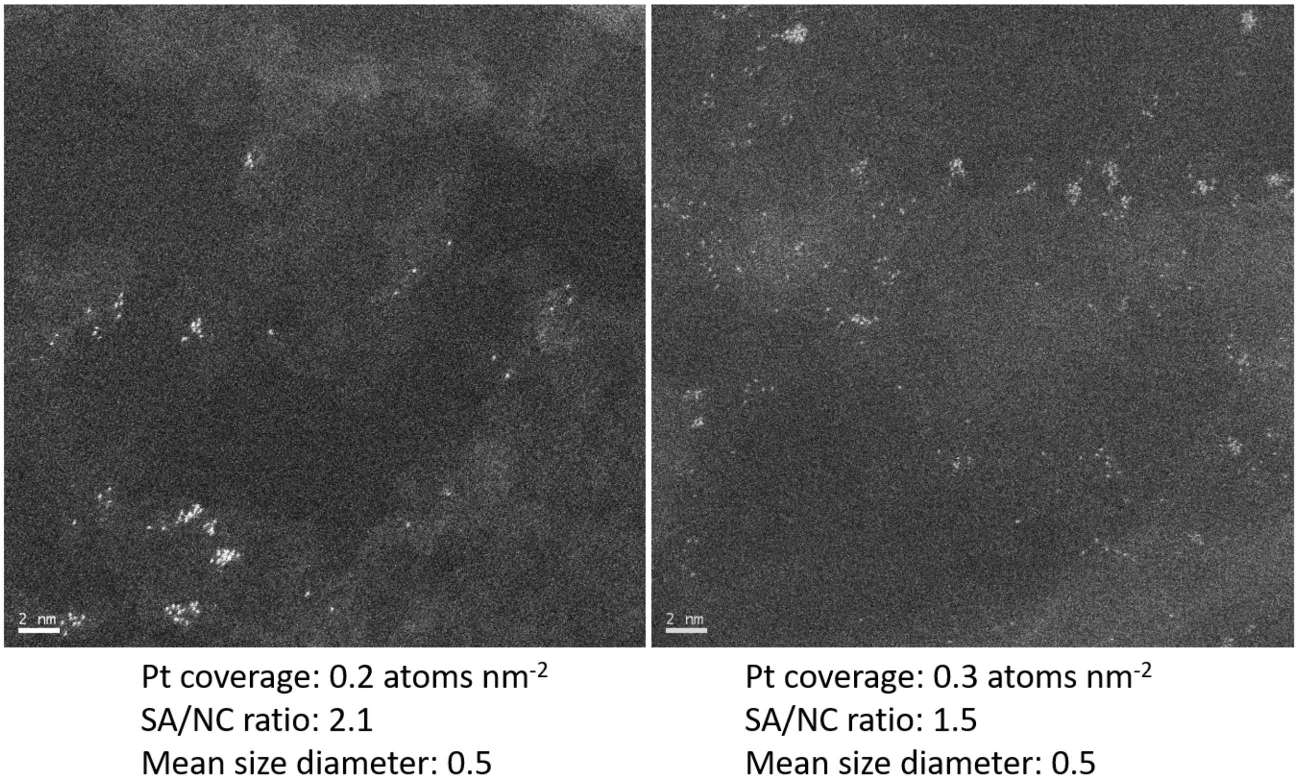

Figure S8: Electron microscopy images of the validation 1 sample used to calculate the Pt coverage onto *h*-BN, SA:NC ratio and NCs mean diameter only using AC-STEM images. Pt coverage onto *h*-BN was found 0.22 ± 0.10 atoms nm^{-2}, SA:NC 1.5 ± 0.5 and mean size diameter 0.6 ± 0.2 nm.

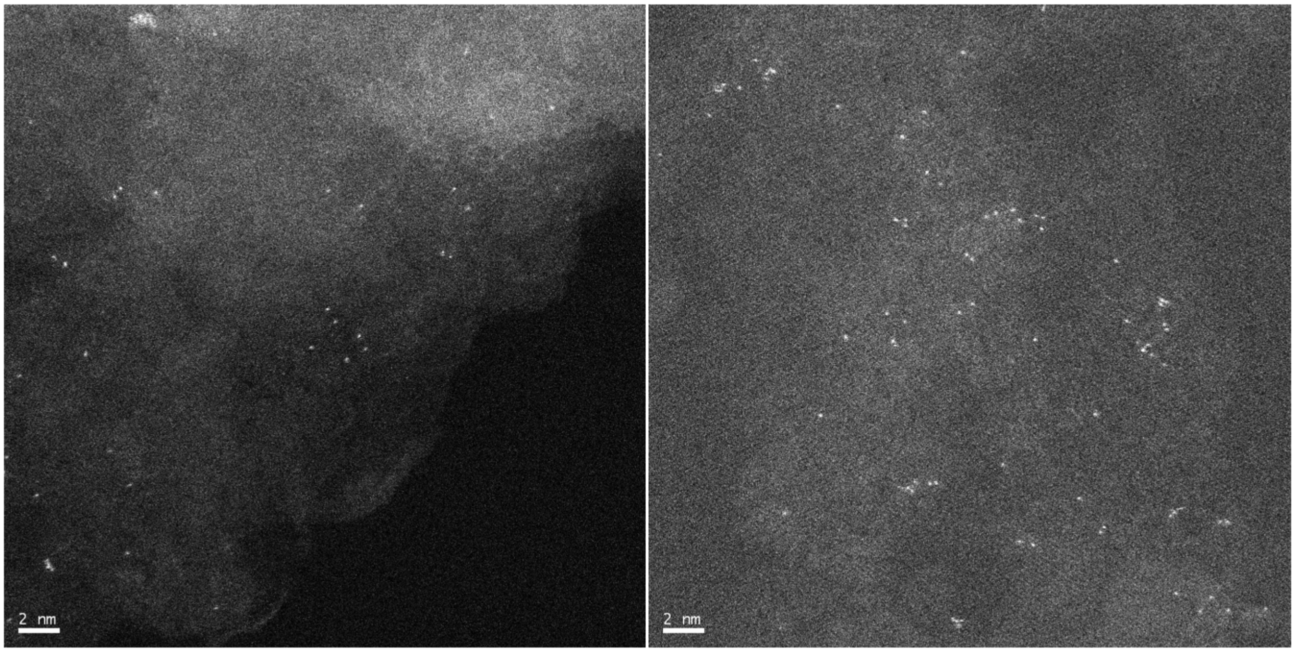

Figure S9: Electron microscopy images of the validation 2 sample used to calculate the Pt coverage onto h-BN, SA:NC ratio and NCs mean diameter only using AC-STEM images. Pt coverage onto h-BN was found 0.05 ± 0.03 atoms nm^{-2}; SA:NC 4.8 ± 2.5 and mean size diameter 0.6 ± 0.1 nm.

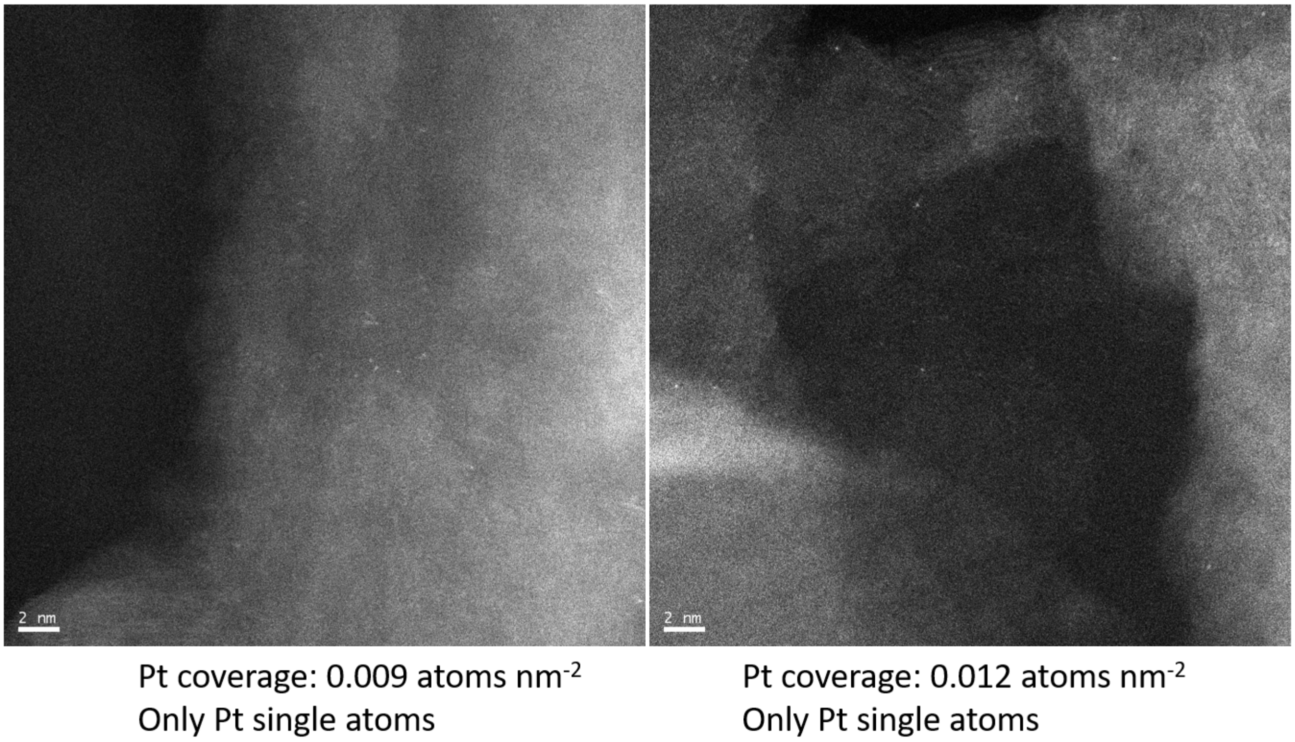

Figure S10: Electron microscopy images of the validation 3 sample used to calculate the Pt coverage onto *h*-BN only using AC-STEM images. These images contain only single atoms. Pt coverage onto *h*-BN was found 0.01 ± 0.01 atoms nm^{-2}.

S2.3 Photoluminescence (PL) measurements

Hexagonal boron nitride (*h*-BN) powder (200 mg) was dispersed in isopropyl alcohol (400 mL) and sonicated for 15 min. The *h*-BN flakes were filtered, dried under room temperature conditions, and placed on a steel plate. Room temperature PL measurements of emission of *h*-BN flakes were carried out using a Renishaw InVia confocal Raman microscope with a 50x objective lens and the excitation laser of 2.71 eV (457 nm). The excitation light and the collected PL signal passed through the same microscope objective, which had the optical axis oriented at the normal incidence angle with respect to the support. The collected PL signal was dispersed through a 300 ln/mm grating and detected by a Peltier-cooled charge-coupled (CCD) camera.

Figure S11 demonstrates a broad PL spectrum in the visible range which comprises several zero-phonon lines (ZPLs) and their phonon sidebands, previously reported in the literature.[7–10] In our setup, the exciton energy is below the bandgap of the material of 5.28 eV (235 nm); it is however above the in-gap donor (-0.6 to -1.5 eV below the conduction band) and acceptor (+1.0 to +1.4 eV above the valence band) states,[8] so those defect levels can be populated resulting in subsequent donor-acceptor radiative recombination. A spike at 2.55 eV (487 nm) corresponds to a Raman scattering of the excitation photon on an optical phonon.[11] The energy of the optical phonon measured at 168 meV in our work is close to the value of 167.9 meV reported in Ref.[11] The shoulder at 2.37 eV (523 nm) and peak at 2.35 (528) eV are donor-acceptor transitions,[8] which can be attributed to the charged boron vacancies.[11] Two peaks, at 2.19 eV (565 nm) and 1.82 eV (682 nm), are also reported as defect-related ZPLs,[7,9,10] and they overlap with the possible phonon replicas of the 2.35 eV ZPL. The shoulder depicted at 610 nm (2.03 eV) is a phonon sideband of 2.19 eV ZPL.[7]

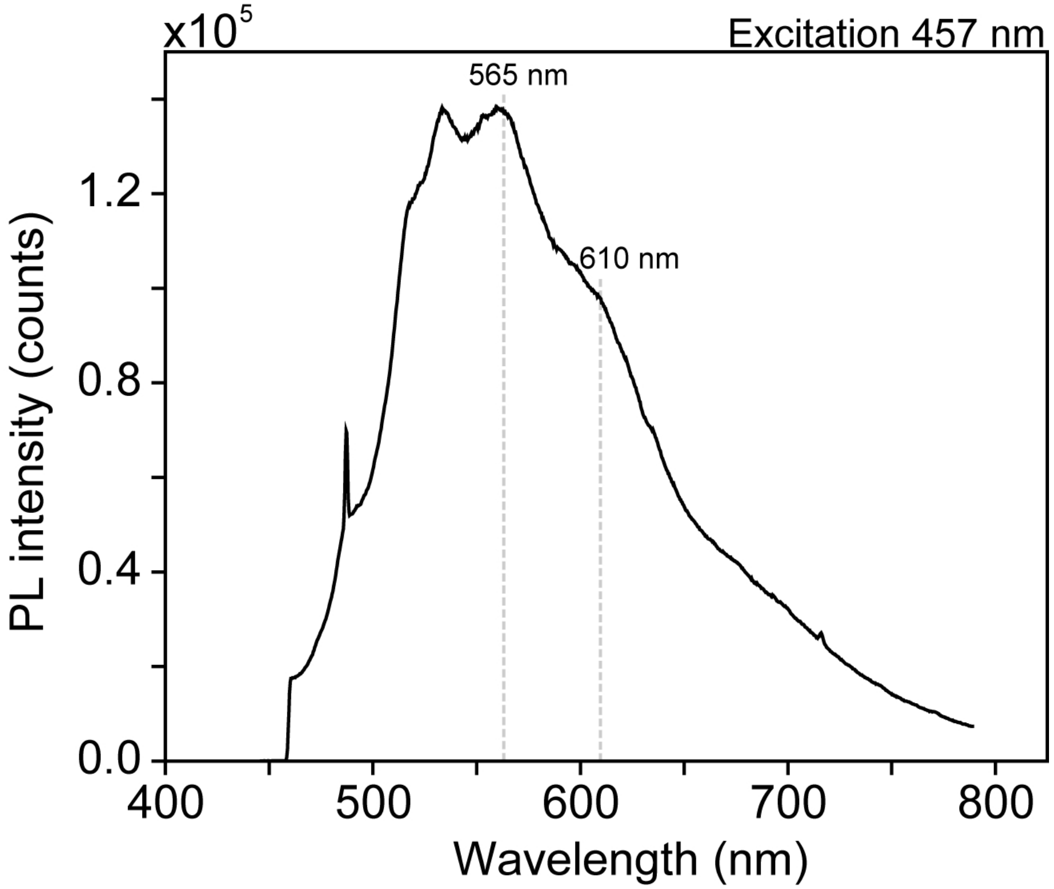

Figure S11: PL measurements of h-BN using a laser power of 0.3 mW, laser spot diameter of 8 μm, 50 accumulations with exposure time of 1 s and laser excitation wavelength of 457 nm.

References

(1) Chakraverty, B. Grain size distribution in thin films–1. Conservative systems. *Journal of Physics and Chemistry of Solids* **1967**, *28*, 2401–2412.

(2) Alcock, C. *CRC Handbook of Chemistry and Physics, 95th Edition*; CRC Press: Boca Raton, Florida, 2015; pp 4–125 – 4–126.

(3) Venables, J. A. Rate equation approaches to thin film nucleation kinetics. *Philosophical Magazine* **1973**, *27*, 697–738.

(4) Yazyev, O. V.; Pasquarello, A. Metal adatoms on graphene and hexagonal boron nitride: Towards rational design of self-assembly templates. *Physical Review B* **2010**, *82*, 045407.

(5) Nie, X.; Li, G.; Jiang, Z.; Li, W.; Ouyang, T.; Wang, J. Co-Solvent Exfoliation of Hexagonal Boron Nitride: Effect of Raw Bulk Boron Nitride Size and Co-Solvent Composition. *Nanomaterials* **2020**, *10*, 1035.

(6) Ramalingam, B.; Mukherjee, S.; Mathai, C. J.; Gangopadhyay, K.; Gangopadhyay, S. Sub-2 nm size and density tunable platinum nanoparticles using room temperature tilted-target sputtering. *Nanotechnology* **2013**, *24*, 205602.

(7) Wang, Q.; Zhang, Q.; Zhao, X.; Luo, X.; Wong, C. P. Y.; Wang, J.; Wan, D.; Venkatesan, T.; Pennycook, S. J.; Loh, K. P.; Eda, G.; Wee, A. T. S. Photoluminescence Upconversion by Defects in Hexagonal Boron Nitride. *Nano Letters* **2018**, *18*, 6898–6905.

(8) Jin, M.-S.; Kim, N.-O. Photoluminescence of Hexagonal Boron Nitride (h-BN) Film. *Journal of Electrical Engineering and Technology* **2010**, *5*, 637–639.

(9) Jungwirth, N. R.; Calderon, B.; Ji, Y.; Spencer, M. G.; Flatté, M. E.; ; Fuchs, G. D.

Temperature Dependence of Wavelength Selectable Zero-Phonon Emission from Single Defects in Hexagonal Boron Nitride. *Nano Letters* **2016**, *16*, 6052–6057.

(10) Reimers, J. R.; Shen, J.; Kianinia, M.; Bradac, C.; Aharonovich, I.; Ford, M. J.; Piecuch, P. Photoluminescence, photophysics, and photochemistry of the V_B^- defect in hexagonal boron nitride. *Physical Review B* **2020**, *102*, 144105.

(11) Jung, S.; Park, M.; Park, J.; Jeong, T.-Y.; Kim, H.-J.; Watanabe, K.; Taniguchi, T.; Ha, D. H.; Hwang, C.; Kim, Y.-S. Vibrational Properties of h-BN and h-BN-Graphene Heterostructures Probed by Inelastic Electron Tunneling Spectroscopy. *Scientific Reports* **2015**, *5*, 16642.
